# Prognostic implications of [¹⁸F]FDG PET and metabolic changes in patients with advanced metastatic neuroendocrine tumors undergoing rechallenge PRRT: final results from a multicenter 10-year survival WARMTH study

**DOI:** 10.7150/thno.123273

**Published:** 2026-01-01

**Authors:** Giulia Santo, Margarida Rodrigues, Diana Paez, Olga Morozova, Levent Kabasakal, Kalevi Kairemo, Chiara Maria Grana, Richard P. Baum, Gianpaolo di Santo, Irene J. Virgolini

**Affiliations:** 1 Department of Nuclear Medicine, Medical University of Innsbruck, Innsbruck, Austria.; 2 Department of Experimental and Clinical Medicine, "Magna Graecia" University of Catanzaro, Catanzaro, Italy.; 3 Nuclear Medicine and Diagnostic Imaging Section, Division of Human Health, International Atomic Energy Agency (IAEA), Vienna, Austria.; 4 Department of Nuclear Medicine, İstanbul University-Cerrahpasa Medical Faculty, İstanbul, Türkiye.; 5 Nuclear Medicine and Theragnostics, Docrates Cancer Center, Helsinki, Finland.; 6 Radiometabolic Therapy Unit, Division of Nuclear Medicine, IRCCS IEO European Institute of Oncology, Milano, Italy.; 7 Molecular Radiotherapy, Curanosticum Wiesbaden-Frankfurt, Germany.; 8 Theranostics Center for Molecular Radiotherapy and Precision Oncology, ENETS Center of Excellence, Zentralklinik Bad Berka, Bad Berka, Germany.

**Keywords:** neuroendocrine tumors, peptide receptor radionuclide therapy, rechallenge, [^18^F]FDG PET, prognosis

## Abstract

**Rationale:** Rechallenge peptide receptor radionuclide therapy (PRRT) is a valid therapeutic option for patients with advanced/metastatic neuroendocrine tumors (NETs) who previously benefited from initial PRRT. In this context, [^18^F]FDG PET may serve as a prognostic marker. This multicenter 10-year survival study aims to evaluate the prognostic implications of [^18^F]FDG PET and PRRT-induced changes in NET patients undergoing rechallenge PRRT.

**Methods:** This retrospective multicenter study included 100 patients (median age: 54 years, range: 29-83) treated with rechallenge PRRT. All patients underwent [^68^Ga]Ga-DOTA-TOC/TATE/NOC and [^18^F]FDG PET/CT prior to the first PRRT period, 3-4 months after PRRT, and every 6-9 months thereafter. Metabolic status and its changes (no change vs. FDG^+^/FDG^-^ vs. FDG^-^/FDG^+^) before the first PRRT period and at each restaging were recorded and correlated to baseline characteristics, time to progression (TTP), and overall survival (OS).

**Results:** In 43 out of 100 patients, the primary tumor site was the pancreas; the liver was involved in more than 90% of patients. Biopsies revealed G1 NET in 16%, G2 NET in 66%, and G3 NET in 18% of cases. Before the first PRRT period, 50% of patients were FDG-positive. Following the first PRRT period, 27 patients exhibited a change in metabolic status: 20 converted to FDG-negative, whereas 7 became FDG-positive. After the second PRRT period, metabolic status changed in 41 patients, with 25 converting to FDG-negative and 16 to FDG-positive. Metabolic status after the first period was significantly correlated with NET grade (*p =* 0.009). The correlation persisted also after rechallenge (*p <* 0.001), suggesting that FDG positivity increased progressively in G3 NET patients (*p =* 0.020). The presence of bone metastases statistically correlated with FDG positivity before (*p <* 0.001) and after (*p =* 0.001) the first PRRT period. Multivariate Cox regression analysis revealed NET G3 and FDG status after the first PRRT course as independent factors for shorter TTP. After a median follow-up time of 117.6 months (range: 38.4-180 months), 37 patients had died. Multivariate Cox regression analysis revealed FDG positivity after the first (*p <* 0.001) and second (*p <* 0.001) periods of PRRT as independent predictors of poor OS.

**Conclusions:** Assessing [^18^F]FDG status before PRRT and during follow-up after treatment enables prediction of TTP and OS, even in patients considered for rechallenge PRRT. Standardizing the use of dual-tracer imaging in patients receiving PRRT seems a valuable approach to improve clinical decision-making in NET patients.

## Introduction

Neuroendocrine tumors (NETs) are a heterogeneous group of diseases originating from neuroendocrine cells, which are widely distributed through the human body. NETs most commonly arise in the gastroenteropancreatic (GEP) system and in the lungs. Based on proliferative activity, measured by the Ki-67 index and/or mitotic rate, they are typically classified into grade 1 (G1), grade 2 (G2), and grade 3 (G3) NETs 1, 2.

Due to the overexpression of somatostatin receptors (SSTR) on their cell surface, NETs have become a paradigm for thera(g)nostics, enabling both imaging and treatment using SSTR-based radiopharmaceuticals 3.

Peptide receptor radionuclide therapy (PRRT) is now a well-established treatment and a standard of care for NET patients 4-7. However, treatment options are limited for patients who experience disease progression after PRRT. In this scenario, retreatment with additional PRRT cycles is considered a viable option for those patients who previously benefited, showing outcomes and safety profiles comparable to the initial treatment 8-11.

Positron emission tomography/computed tomography (PET/CT) plays a critical role in assessing tumor biology, guiding treatment decisions, and refining prognostic evaluation. Nevertheless, the routine use of [^18^F]fluorodeoxyglucose ([^18^F]FDG) PET/CT remains debated. Current European Neuroendocrine Tumor Society (ENETS) and European Society for Medical Oncology (ESMO) guidelines recommend [^18^F]FDG PET selectively, especially in cases with Ki-67 >10% (i.e., high G2 and G3 NET) 12,13. However, clinical evidence highlights the substantial heterogeneity of NET within the overall tumor burden 14. Spatiotemporal heterogeneity, in fact, significantly influences both disease progression and response to therapy 15. It is known that different tumor clones with varying levels of proliferation and differentiation may coexist at diagnosis or emerge during disease evolution 16-18.

A dual-tracer imaging approach, combining [^18^F]FDG and [^68^Ga]Ga-DOTA-labeled-somatostatin analogs (SSA) PET/CT, has been proposed to more comprehensively assess tumor behavior 19,20.

However, in patients undergoing rechallenge PRRT, the role of metabolic status as assessed by [^18^F]FDG PET and changes observed over PRRT periods remains only partially explored. A small preliminary study on 40 patients with advanced NET undergoing rechallenge PRRT showed that FDG-positive (FDG^+^) patients after rechallenge had a worse outcome compared to FDG-negative (FDG^-^) patients (median overall survival: 96 months vs. 145.5 months) 21. Nonetheless, the impact of longitudinal [^18^F]FDG PET assessment remains unclear.

This multicenter retrospective 10-year survival study aims to evaluate the prognostic implications of [^18^F]FDG PET/CT and PRRT-induced metabolic changes over time in patients treated with rechallenge PRRT.

## Materials and Methods

### Patient identification

A multicenter retrospective study was conducted across five centers in Austria, Italy, Germany, Turkey, and Finland. Adult patients diagnosed with unresectable or metastatic SSTR-positive NETs who experienced disease progression after completing an initial course of PRRT and subsequently underwent rechallenge PRRT were analyzed.

To be included in the study, all patients were required to have undergone both [⁶⁸Ga]Ga-DOTA-SSA and [¹⁸F]FDG PET/CT scans prior to the first PRRT period, 3-4 months after PRRT, and every 6-9 months thereafter. No anticancer therapy, including PRRT, was administered between these scans.

Patients were treated with DOTA-conjugated somatostatin analogs (DOTA-TOC, DOTA-TATE) radiolabeled with ^177^Lu or ^90^Y, according to European Association of Nuclear Medicine (EANM) guidelines 22.

Patient- and disease-related data were retrospectively collected from hospital electronic medical records. Clinical and pathological variables included age at diagnosis, gender, primary NET site, histological grade, and sites of metastasis.

The study was conducted in accordance with the ethical standards of the 1964 Declaration of Helsinki and later amendments and approved by the Ethics Committee of the Medical University of Innsbruck (EK Nr: 1195/2018) and by local ethics committees at the participating centers.

### Imaging analysis

Image data were acquired using PET/CT scanners at each institution, and the images were analyzed with commercially available software that allowed for review of PET, CT, and fused imaging data. Table S1 provides detailed information on the scanners and software used for the study at each center. Scans were analyzed subjectively by visual interpretation on dedicated nuclear medicine reporting workstations. Interpretation was performed by board-certified nuclear medicine physicians with more than 10 years of experience in PET reading. For each time point, [⁶⁸Ga]Ga-DOTA-SSA and [¹⁸F]FDG PET/CT scans were displayed simultaneously in transverse, coronal, and sagittal planes, accompanied by a maximal-intensity projection of the PET data, with each image set anatomically coregistered.

A positive finding on [⁶⁸Ga]Ga-DOTA-SSA PET was defined as a focal area of increased tracer uptake that could not be explained by physiologic distribution or that exceeded background organ activity, particularly when corresponding to an abnormal structure on CT. Lesion(s) showing distinct higher uptake than liver activity were classified as positive for enhanced SSTR expression and therefore considered suggestive of malignancy 23, 24.

An [¹⁸F]FDG PET scan was considered positive if at least one [¹⁸F]FDG-avid lesion was detected. Positive [¹⁸F]FDG PET findings were defined as abnormal FDG accumulation on PET, particularly focal uptake, that could not be attributed to physiologic biodistribution, inflammation, or other non-malignant processes, especially when corresponding to a tumor lesion identified on CT. No fixed threshold for [¹⁸F]FDG uptake intensity was applied, as interpretation relied on the contrast between the lesion and surrounding tissue and took into account potential differences in [¹⁸F]FDG avidity across tumor lesions 25.

[¹⁸F]FDG status (FDG^+^ vs. FDG^-^) was recorded prior to the first PRRT and after treatment. Accordingly, metabolic changes during the follow-up period after the first and second PRRT courses compared to the FDG status before the start of the first PRRT period were reported as follows: i) no change in FDG status, ii) shift from FDG-positive to FDG-negative status (FDG^+^/FDG^-^), iii) shift from FDG-negative to FDG-positive status (FDG^-^/FDG^+^).

### Tumor response assessment

According to oncological guidelines 12, 13, tumor response to PRRT was assessed either by contrast-enhanced CT and/or magnetic resonance imaging (MRI), as appropriate, and categorized following RECIST 1.1 criteria into complete remission (CR), partial remission (PR), stable disease (SD), or progressive disease (PD). In addition, both [⁶⁸Ga]Ga-DOTA-SSA and [¹⁸F]FDG PET/CT were used (i) for monitoring NET lesions that were only visualized on molecular imaging (e.g., small, non-enlarged nodes, or bone metastases), and/or (ii) to confirm equivocal anatomical findings 26, 27.

Restaging was performed 3-4 months after each PRRT period and every 6-9 months thereafter. Patients were followed up for stable disease or remission (complete or partial) until disease progression before the rechallenge. The final disease course was determined based on the response assessment performed after the second PRRT period.

### Statistical data analysis

SPSS software version 30.0 (SPSS Inc., Chicago, IL, USA, and LEAD Technologies, Charlotte, NC, USA) and GraphPad Prism version 10.5 (GraphPad Software, Boston, MA, USA) were used for statistical analysis. The associations between baseline variables and [^18^F]FDG PET results were assessed using Pearson's chi-square test for categorical variables and Student's t-test for continuous variables.

Time to progression (TTP) was defined as the interval between the last cycle of the first PRRT period and disease progression. Overall survival (OS) was calculated from the date of initial histological diagnosis or the first [^68^Ga]Ga-DOTA-TOC/TATE/NOC PET/CT examination to the date of death or, for surviving patients, the last day of follow-up. Univariate and multivariate Cox regression analyses were used to investigate prognostic factors affecting TTP and OS. The multivariate analysis was performed by entering the following variables into the model: primary tumor site (i.e., other primary vs. pancreatic NETs), presence of lymph node metastases, presence of bone metastases, NET grade, and FDG status before and after PRRT. Survival probability was estimated using the Kaplan-Meier method. The log-rank test was applied to compare survival distributions between groups. In all analyses, a *p*-value < 0.05 was considered statistically significant.

## Results

### Patient characteristics

One hundred patients were included in the study (64 males and 36 females; median age 54 years, range: 29 - 83 years). Table 1 provides the patients' baseline characteristics. In 43% of patients, the primary tumor site was the pancreas, followed by the midgut (38%). Among patients, 16 had G1 NET, 66 had G2 NET, and 18 had G3 NET. The liver was involved in more than 90% of cases, followed by lymph nodes (40%) and bone (30%). Most patients received [^177^Lu]Lu-DOTA-TATE during both initial and rechallenge PRRT (78% and 89%, respectively).

### [^18^F]FDG status and correlation with clinical-pathological features

**Figure 1:**
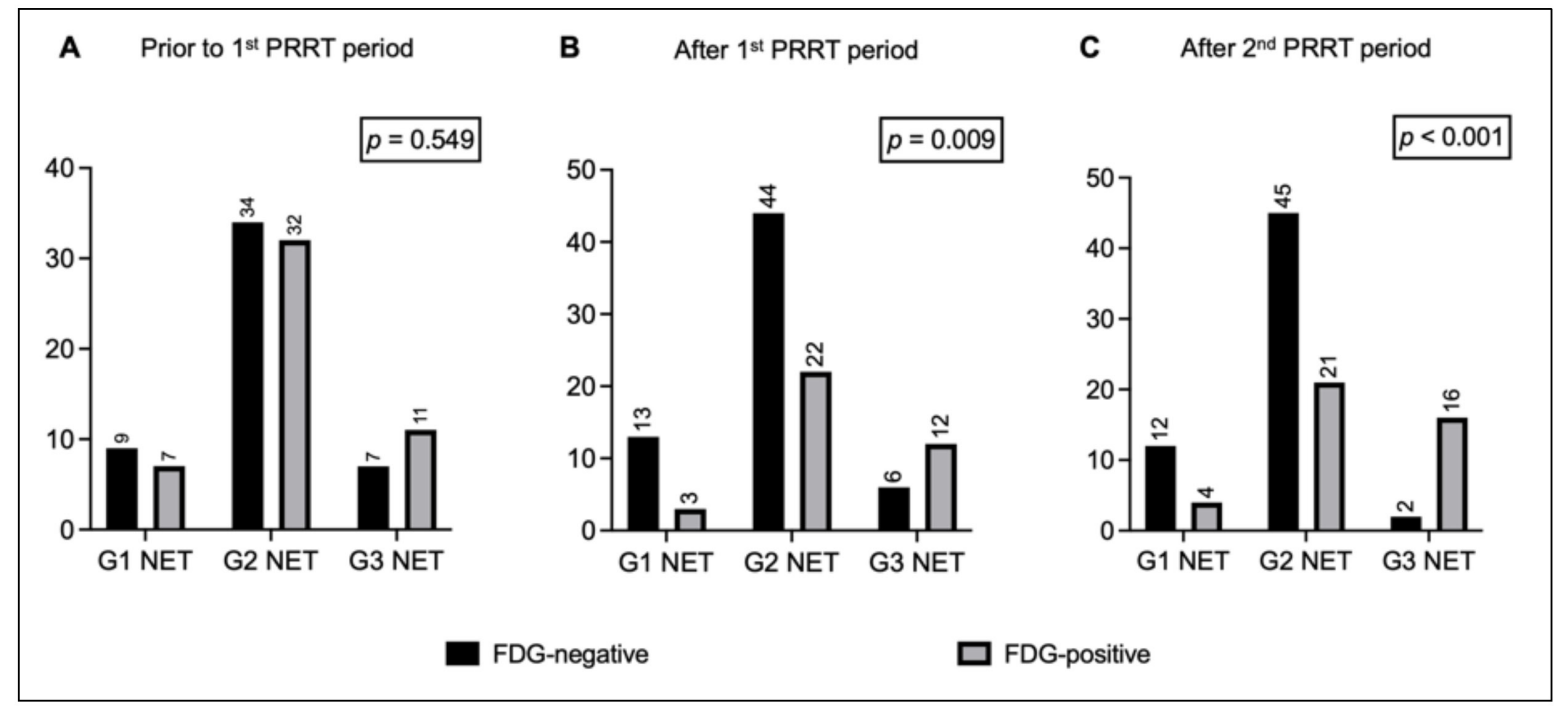
Bar charts comparing distribution of FDG-negative and FDG-positive patients with G1 NET, G2 NET, and G3 NET (A) prior to the 1^st^ PRRT period, (B) after the 1^st^ PRRT period, and (C) after the 2^nd^ PRRT period. Legend: FDG = [^18^F]fluorodeoxyglucose; NET = neuroendocrine tumor; PET = positron emission tomography; PRRT = peptide receptor radionuclide therapy.

Prior to the first PRRT period, 50 out of 100 patients showed [^18^F]FDG PET positivity. Among them, 7 had G1 NET, 32 had G2 NET, and 11 had G3 NET. NET grade did not correlate with FDG positivity before treatment (Figure 1A).

**Figure 2:**
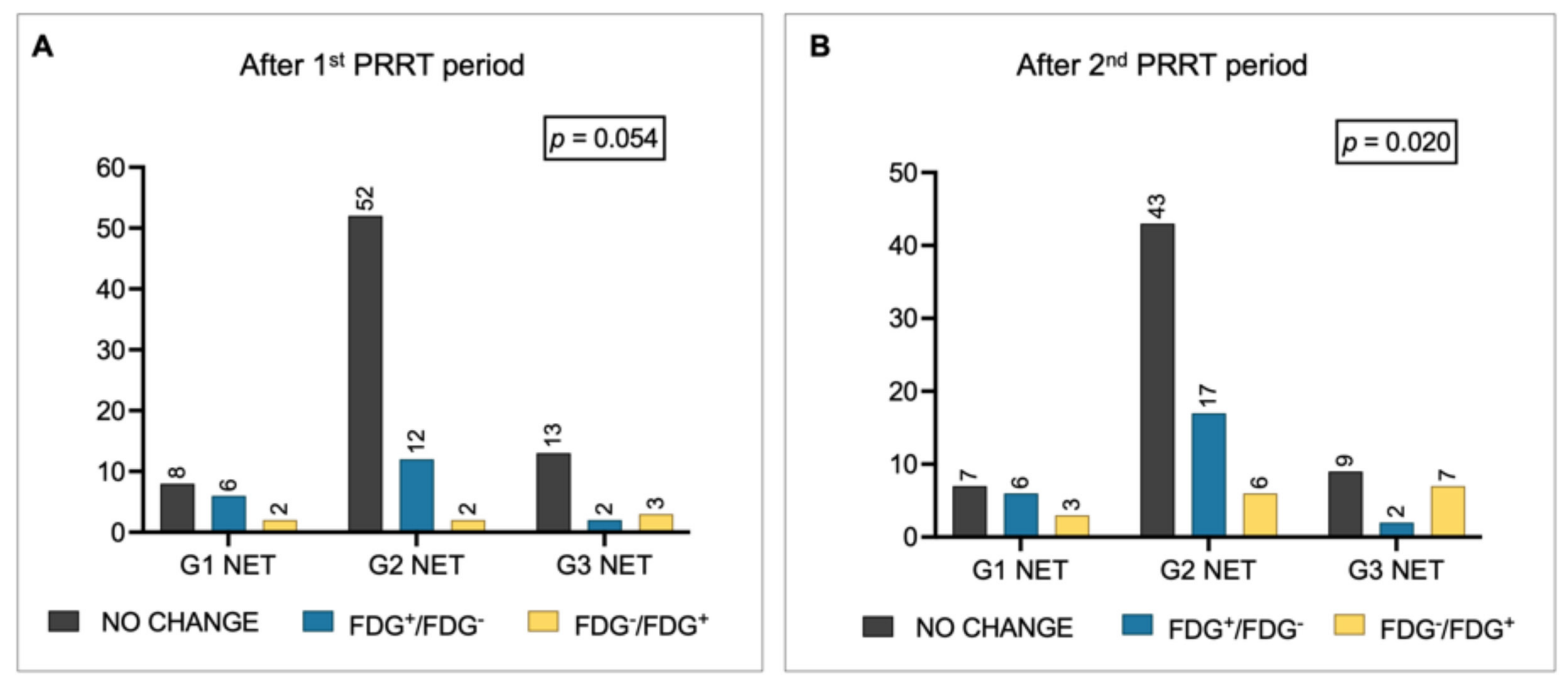
Metabolic changes in FDG status among patients with G1, G2, and G3 NET (A) after the 1^st^ PRRT period and (B) after the 2^nd^ PRRT period. Legend: FDG = [^18^F]fluorodeoxyglucose; NET = neuroendocrine tumor; PET = positron emission tomography; PRRT = peptide receptor radionuclide therapy.

Overall, 27% of patients change their metabolic status after the first PRRT period, 41% after rechallenge. Specifically, after the first period, 37 out of 100 patients were FDG-positive, while 63 were FDG-negative: 20 patients became metabolically negative, 7 became FDG^+^, and the remaining 73 patients did not change their metabolic status (i.e., 43 FDG^-^ and 30 FDG^+^). FDG positivity after the first PRRT significantly correlated with NET grade (FDG-positive: 19% of G1 NET, 33% of G2 NET, and 67% of G3 NET, *p* = 0.009; Figure 1B). There was a trend toward significance for G1/G2 patients shifting from FDG^+^ to FDG^-^ compared with G3 NET patients (*p* = 0.054; Figure 2A).

After the second PRRT period, 41 out of 100 patients were FDG-positive, while 59 were FDG-negative: 25 patients converted to FDG^-^, 16 converted to FDG^+^, and the remaining 59 patients did not change their metabolic status (i.e., 34 FDG^-^ and 25 FDG^+^). The correlation between NET grade and FDG status persisted after rechallenge (FDG-positive: 25% of G1 NET, 32% of G2 NET, 89% of G3 NET, *p* < 0.001; Figure 1C), suggesting that the proportion of patients who converted to FDG^+^ increased progressively in G3 NET patients after rechallenge PRRT (*p* = 0.020). Conversely, among G2 NET patients, there was an increase (+5 patients) in those who became FDG^-^ after rechallenge compared with the first course (Figure 2B).

Furthermore, the presence of bone metastases was significantly associated with FDG positivity before (*p* < 0.001) and after (*p* = 0.001) the first PRRT course, but the correlation was not observed after the second period (Figure S1). Additionally, there was a trend toward higher FDG positivity before the first PRRT in patients with pancreatic NETs compared with those with other primary sites (60% vs. 42%, *p* = 0.069) (Figure S2). No other clinical or pathological features correlated with FDG positivity before or after the treatment (data not shown).

### Predictors of TTP after the first PRRT period

**Figure 3:**
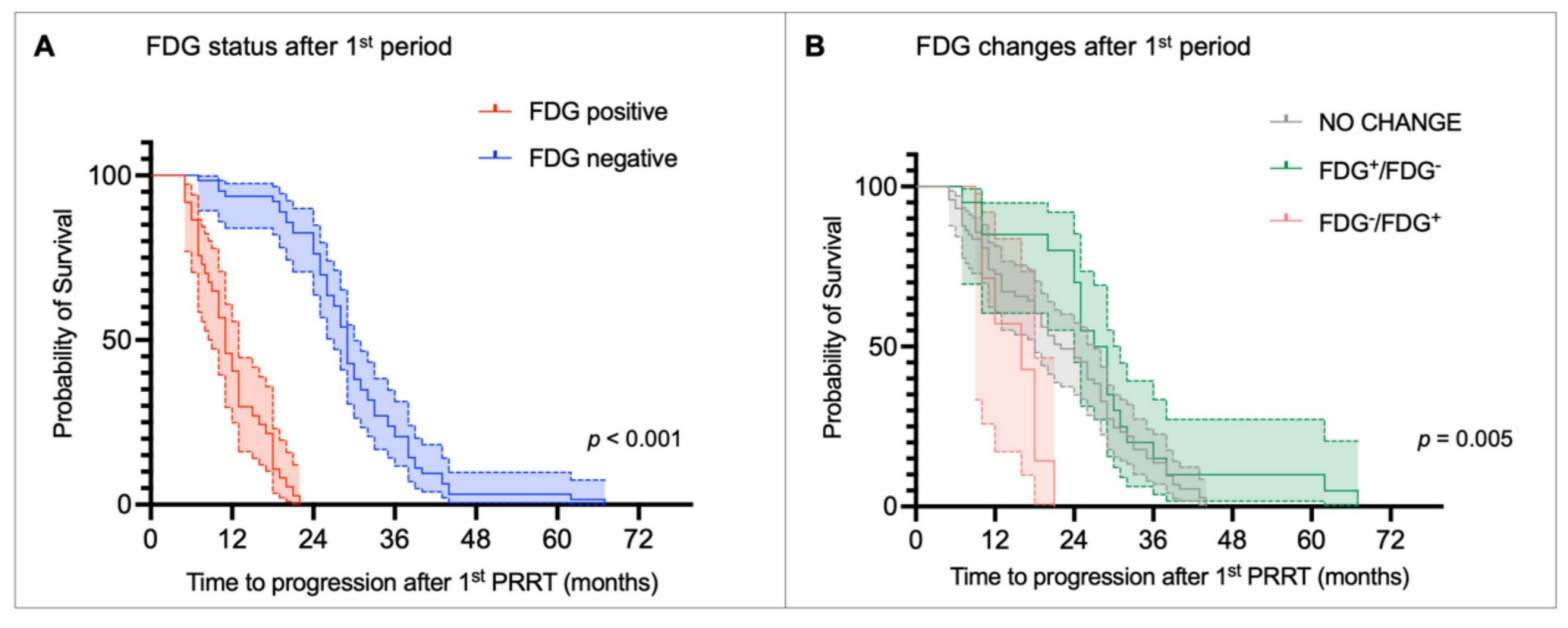
Kaplan-Meier curves for time to progression (TTP) stratified by (A) [^18^F]fluorodeoxyglucose (FDG) status after the first peptide receptor radionuclide therapy (PRRT) period and (B) metabolic changes before and after the first PRRT period.

The median TTP after the first PRRT period was 24 months (range: 5-67 months). Table 2 summarizes the results of the univariate and multivariate Cox regression analyses for TTP. The univariate analysis showed that the presence of bone metastases (HR 1.861, *p =* 0.007), as well as FDG positivity before (HR 1.972, *p <* 0.001) and after the first PRRT period (HR 18.921, *p <* 0.001) was significantly associated with shorter TTP. Consistently, patients with no metabolic changes (HR 0.353, *p* = 0.013) or converted from FDG-positive to FDG-negative (HR 0.243, *p =* 0.002) had a lower risk of progression compared with those who became FDG-positive after the first PRRT period. In addition, G1 NET (HR 0.230, *p <* 0.001) and G2 NET (HR 0.338, *p <* 0.001) were linked to a reduced risk of progression compared with G3 NET patients. In the multivariate analysis, only NET grade and FDG status after the first PRRT retained independent prognostic significance. Specifically, G3 NET was significantly associated with shorter TTP compared to G1 NET (HR 0.374, *p =* 0.014) and G2 NET (HR 0.448, *p =* 0.007). However, FDG-positive status after the first PRRT remained the strongest predictor of early progression (HR 15.549, *p <* 0.001). Kaplan-Meier curves and the log-rank test confirmed that FDG status was able to stratify TTP. FDG-negative patients after the first PRRT had a median TTP of 29.0 months (95% CI: 27.6-30.4 months) compared to 11.0 months (95% CI: 9.0-13.0 months) in the FDG-positive group (*p <* 0.001, Figure 3A). Consistently, patients who became FDG-positive after the first PRRT period showed the shortest median TTP compared with those whose FDG status remained unchanged or who converted to FDG-negative (median TTP: FDG^-^/FDG^+^ = 16 months vs. no change = 22 months vs. FDG^+^/FDG^-^ = 27 months; *p* < 0.005; Figure 3B).

Interestingly, FDG positivity before the first PRRT was significantly associated with shorter TTP in the univariate analysis (HR 1.972, *p* < 0.001). However, the variable was not identified as an independent predictor of progression by the multivariate model. The Kaplan-Meier curve of TTP according to FDG status before the first PRRT period is shown in Figure S3.

### Impact of [^18^F]FDG PET after the first PRRT period on the final disease course after rechallenge

**Figure 4:**
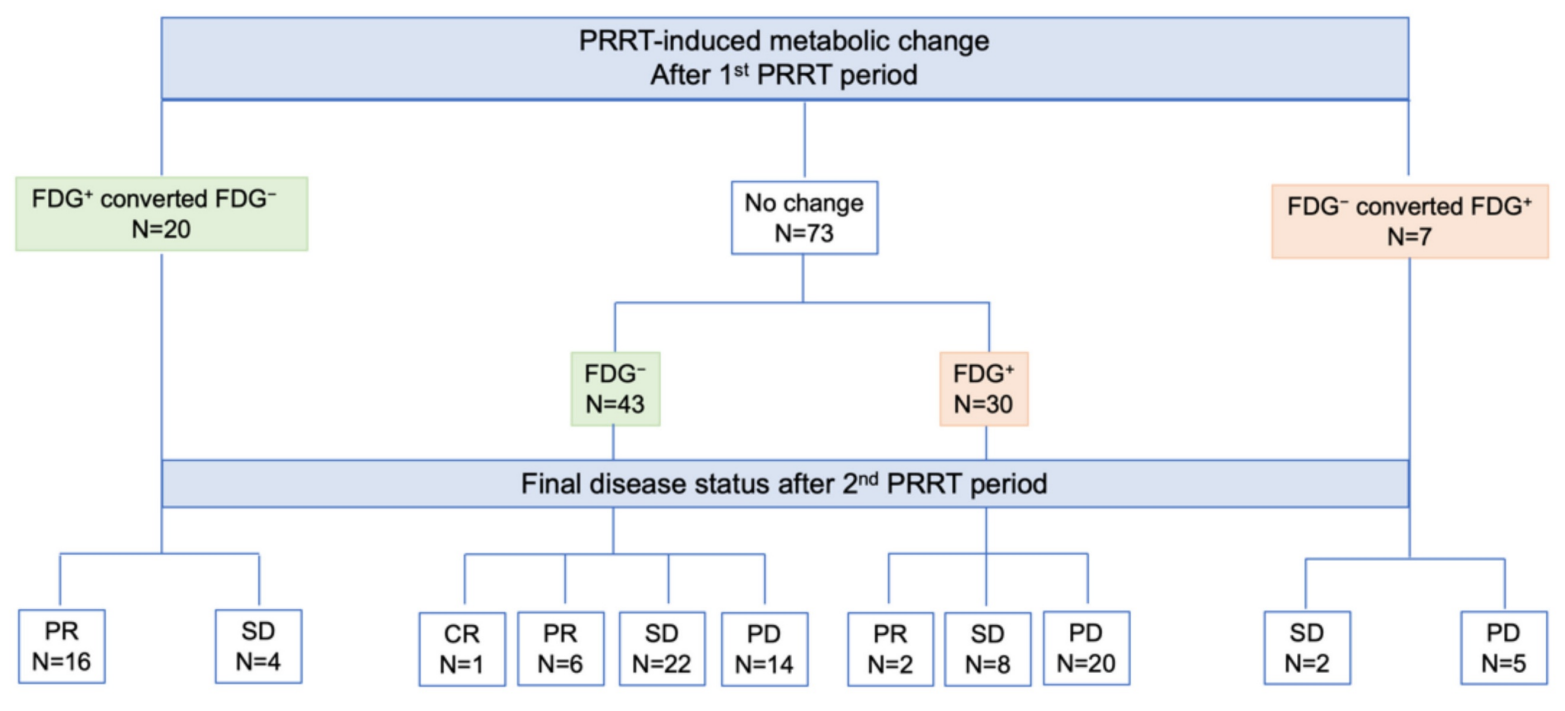
Schematic representation of response after the 2^nd^ PRRT period according to [^18^F]FDG status and PRRT-induced changes after the 1^st^ PRRT period. Legend: CR = complete remission; FDG = [^18^F]fluorodeoxyglucose; NET = neuroendocrine tumor; PD = progressive disease; PET = positron emission tomography; PR = partial remission; PRRT = peptide receptor radionuclide therapy; SD = stable disease.

According to imaging-based restaging after rechallenge PRRT, one patient achieved CR, 24 showed PR, 36 had SD, and 39 experienced PD. Figure 4 illustrates the distribution of [¹⁸F]FDG status and its changes after the first PRRT period in relation to the final disease course after the second PRRT period. Patients who were FDG-positive or converted from FDG-negative to FDG-positive were significantly more likely to progress after rechallenge PRRT compared with those who remained or converted to FDG-negative (68% vs. 22%, *p* < 0.001). Among the 14 patients who were FDG-negative before rechallenge but experienced disease progression, 9 (64%) converted to FDG-positive after rechallenge PRRT. Notably, none of the 20 patients who shifted from FDG-positive to FDG-negative progressed after rechallenge, and a high remission rate of 80% (16 out of 20 patients) was observed in this group.

### Prognostic impact of [^18^F]FDG PET on survival

After a median follow-up of 117.6 months (range: 38.4-180 months), 37 out of 100 patients had died. The median OS for the entire cohort was 156.0 months (95% CI: 139.2-172.8 months).

The univariate Cox regression analysis showed that patients with G3 NET had a significantly shorter OS compared with those with G1 NET (HR 0.123, *p* = 0.001) and G2 NET (HR 0.207, *p <* 0.001). FDG positivity after the first (HR 39.383, *p <* 0.001) and after the second (HR 14.580, *p <* 0.001) PRRT period was associated with poor OS. Consistently, patients who shifted to FDG-positive after the first or the second PRRT course had the shortest OS compared with those whose FDG status remained unchanged or who became FDG-negative. In the multivariate analysis, the presence of lymph node metastases emerged as an independent predictor of longer OS (HR 0.223, *p =* 0.001). Conversely, FDG positivity after the first (HR 27.963; *p <* 0.001) and second (HR 10.321, *p <* 0.001) PRRT periods was confirmed as an independent predictor of poor OS. Interestingly, the G3 NET grade was not identified as an independent predictor of poor OS in the multivariate analysis. Table 3 summarizes the results of the univariate and multivariate Cox regression analyses for OS.

**Figure 5:**
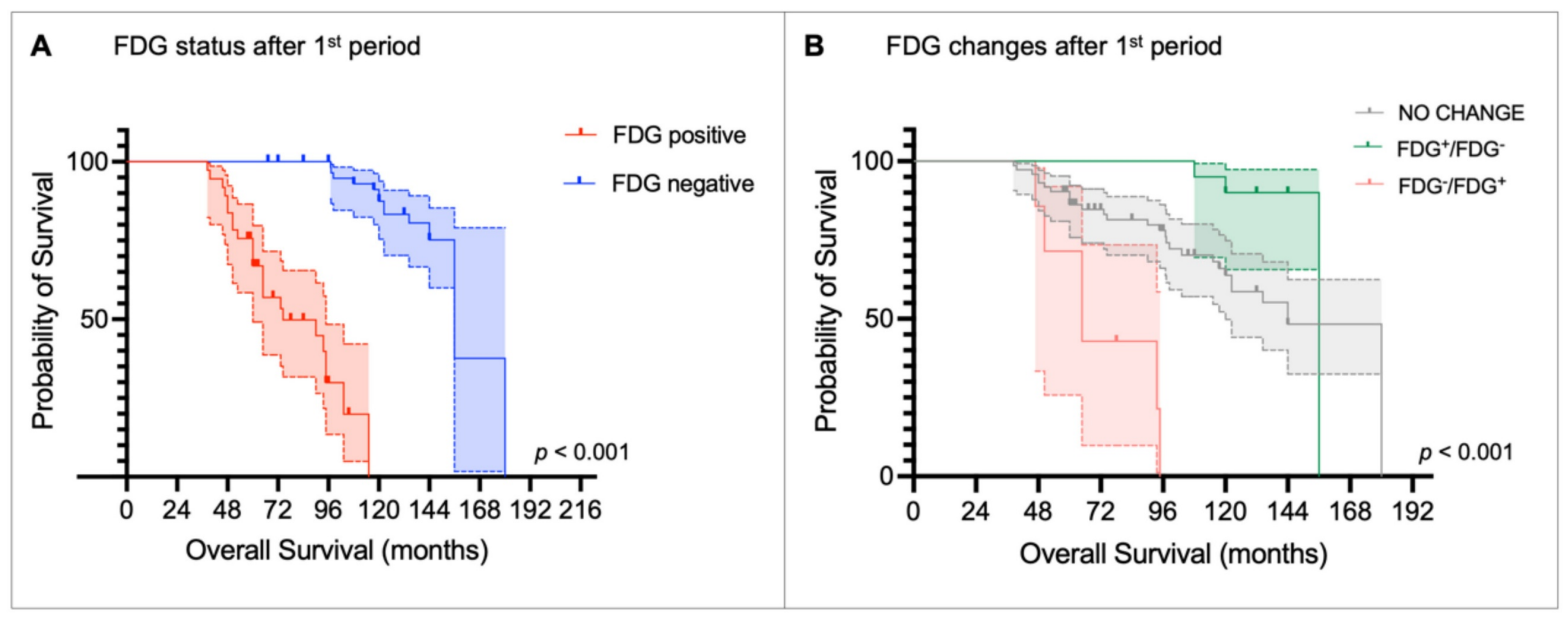
Kaplan-Meier curves for overall survival stratified by (A) [^18^F]fluorodeoxyglucose (FDG) status after the first peptide receptor radionuclide therapy (PRRT) period and (B) metabolic changes before and after the first PRRT period.

**Figure 6:**
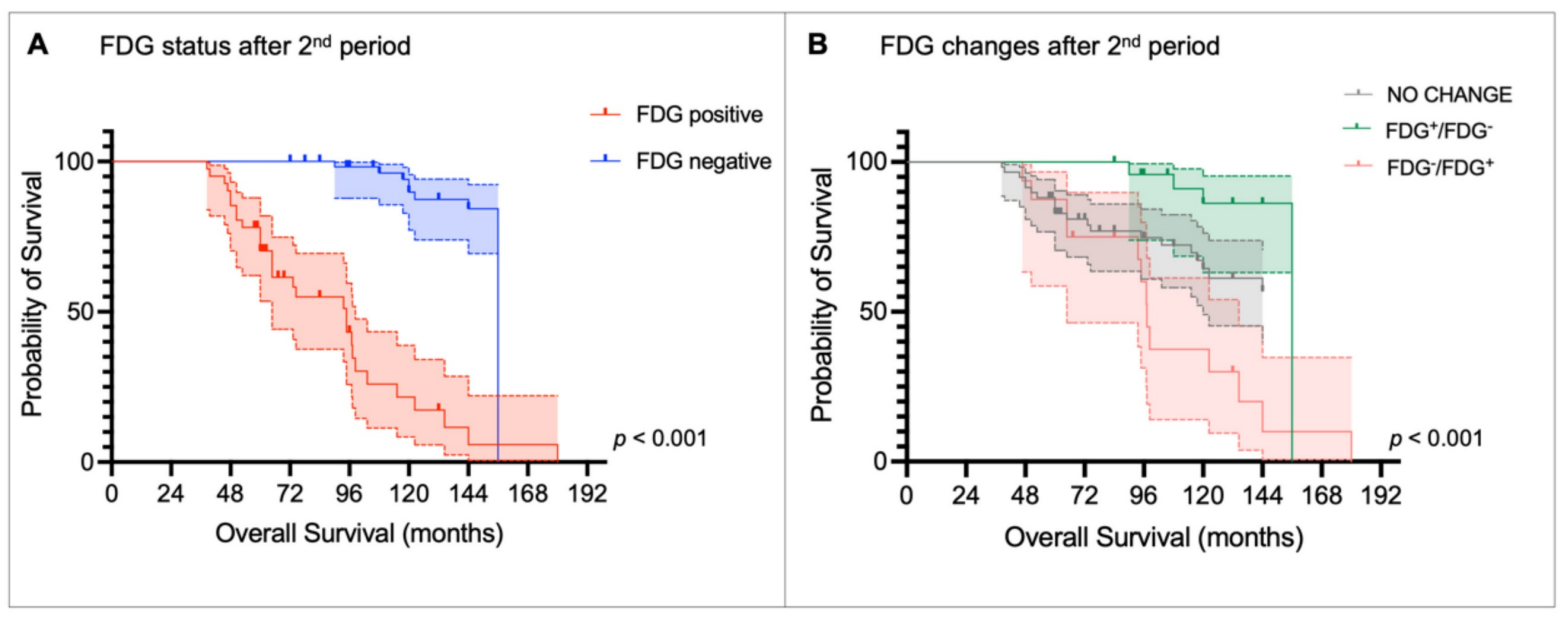
Kaplan-Meier curves for overall survival stratified by (A) [^18^F]fluorodeoxyglucose (FDG) status after the 2^nd^ peptide receptor radionuclide therapy (PRRT) period and (B) metabolic changes after the 2^nd^ PRRT period compared to FDG status before the 1^st^ PRRT period.

Kaplan-Meier curves and the log-rank test confirmed that FDG status after the first PRRT period was able to stratify OS (median OS: FDG^+^ = 74.4 months [95% CI: 38.3-110.5] vs. FDG^-^ = 156.0 months [95% CI: 139.3-172.8], *p <* 0.001; Figure 5A), as it was FDG status after the second PRRT (median OS: FDG^+^ = 94.8 months [95% CI: 69.8 -119.8] vs. FDG^-^ = 156 months [95% CI: NA], *p <* 0.001; Figure 6A). Patients who became FDG-positive after the first PRRT course had the worst median OS compared to those whose FDG status did not change or those who became FDG-negative (median OS: FDG^-^/FDG^+^ = 64.8 months vs. FDG no change = 144.0 months vs. FDG^+^/FDG^-^ = 156.0 months;* p <* 0.001, Figure 5B), with the latter group showing the longest OS. A similar trend was observed after the second PRRT course (median OS: FDG^-^/FDG^+^ = 97.2 months vs. FDG^+^/FDG^-^ = 156.0 months vs. FDG no change = not reached;* p <* 0.001, Figure 6B).

## Discussion

This retrospective multicenter study involving 100 NET patients who underwent rechallenge PRRT evaluated the prognostic significance of [^18^F]FDG PET/CT over two PRRT periods, within a median survival time of more than 10 years.

The role of dual-tracer imaging in NET patients remains a topic of ongoing debate. In fact, although existing evidence has already suggested [^18^F]FDG PET/CT as a powerful predictor of disease biology and prognosis 28, 29, further data are needed to clarify the optimal timing and the clinical context in which [^18^F]FDG PET/CT should be performed.

To the best of our knowledge, this is the first study to explore the prognostic role of [^18^F]FDG PET and the impact of the metabolic changes after treatment in a large cohort of patients undergoing rechallenge PRRT.

One of the first findings of the study was the proportion of patients with G1 and G2 NET who presented with at least one FDG-positive lesion before starting PRRT: 7/16 (44%) of G1 and 32/66 (49%) of G2 patients, respectively. These findings are consistent with previous reports showing FDG positivity even in lower-grade NETs 21, 30, 31, emphasizing the need for a comprehensive understanding of tumor behavior before treatment. In addition, this observation leads to an interesting result of our study: NET grade was not initially correlated with FDG status before the first PRRT cycle, but the correlation became more evident across treatment periods. Although partially inconsistent with previous studies that found an association between FDG positivity and higher-grade NET 28, the evolving correlation observed during treatment may better reflect the dynamic tumor biology and the impact of PRRT on FDG-avid lesions across different NET grades. Overall, 27% of patients change their metabolic status after the first PRRT period, 41% after rechallenge. Specifically, as shown in Figure 1, patients with G1/G2 NET were more likely to convert to FDG-negative status after treatment compared to those with G3 NET. This metabolic modulation induced by PRRT has been reported by other authors in smaller cohorts of patients 21, 32, 33. Although not fully understood, the 'crossfire effect' of beta-minus particles may partially explain this metabolic shift. This phenomenon enables damage to nearby cells even if they are not directly targeted, which is particularly useful in highly heterogeneous tumors 34.

Another finding of the study was the significant association between FDG positivity and the presence of bone metastases. Skeletal involvement is traditionally considered a late manifestation in the natural history of NET 35, likely reflecting the advanced disease stage in our cohort. Although the presence of bone metastases was associated with a shorter TTP, this factor did not appear to affect OS.

In contrast, FDG status and its temporal changes after treatment emerged as strong predictive biomarkers in relation to TTP after the first PRRT. Namely, both G3 NET grade and FDG positivity after the first PRRT were identified as independent prognostic factors for shorter TTP. Interestingly, FDG positivity before the first PRRT period was significant in the univariate analysis but lost significance in the multivariate model, suggesting that dynamic changes in FDG status after PRRT may be more prognostically relevant than a single time-point assessment performed before the start of PRRT. This was further supported by OS analysis, where FDG status before the start of PRRT was not associated with OS. Given the long median OS in our cohort (~13 years), this finding aligns with the study by Paganelli et al. 36, which suggested that the prognostic value of [^18^F]FDG PET/CT may reduce over time (i.e., after 10 years). This result also highlights the importance of repeated [^18^F]FDG PET/CT scans during treatment to accurately stratify patients over time.

One of the most significant findings emerged from the analysis of the correlation between [^18^F]FDG status and PRRT-induced changes after the first treatment period, in relation to the final response to rechallenge PRRT. Specifically, patients who were FDG-positive or converted from FDG-negative to FDG-positive had a significantly higher risk of disease progression after rechallenge PRRT compared to those who remained or became FDG-negative (67.6% vs. 22%, *p* < 0.001). Interestingly, none of the 20 patients who converted from FDG-positive to FDG-negative progressed after rechallenge, and this group showed a high remission rate of 80%. These findings may have practical implications, as [^18^F]FDG PET/CT could serve as a valuable tool in guiding treatment strategies, helping to identify patients more likely to benefit from a second PRRT period, or conversely, those who may require alternative therapeutic approaches.

The survival analysis further corroborated previous findings and further highlighted the prognostic value of repeated [^18^F]FDG PET/CT across treatment periods. FDG status after the first and second PRRT courses was confirmed as an independent prognostic factor for OS. In contrast, G3 NET did not retain independent prognostic significance for OS in the multivariate analysis, particularly when compared with G2 NET patients. These results support the data reported by Binderup et al. 19, who suggested [^18^F]FDG PET as a stronger prognostic marker than NET grade. Consistently, our survival analysis confirmed the significant prognostic impact of PRRT-induced changes in FDG status over time: patients who became FDG-positive during PRRT had the poorest prognosis, whereas those with stable FDG status or conversion to FDG-negative showed more favorable outcomes. This result aligns with the findings of Nilica et al. 37, who showed that stable FDG uptake after PRRT was associated with favorable outcomes. In contrast, among patients with progressive disease who died following PRRT, FDG uptake increased from 41% to 82% between the first and last follow-up.

Therefore, our findings support the use of [^18^F]FDG PET/CT as a powerful prognostic tool for risk stratification over time. Identifying patients with poor prognosis after PRRT may allow for early adjustments in treatment strategy 38, guiding them toward alternative therapies and potentially improving survival outcomes 39-41.

Our study has several limitations that should be acknowledged. Beyond its retrospective design, the use of visual assessment alone may have limited the evaluation of overall tumor burden, restricting further quantitative analysis. Although metabolic semiquantitative parameters such as metabolic tumor volume (MTV) and total lesion glycolysis (TLG) have been reported as prognostic markers in advanced NET patients 42, we chose visual analysis to minimize inter- and intra-center variability during the long follow-up period. However, heterogeneity across centers in terms of PRRT radiopharmaceuticals, administered activity, and number of treatment cycles represents an additional limitation of the study. Furthermore, the study population consisted of patients undergoing third-line systemic treatment (PRRT rechallenge) with a different distribution of NET grades and the highest prevalence of GEP-NET, with liver involvement in more than 90% of cases. This selection bias could limit the reproducibility and generalizability of our findings. Lastly, future research should incorporate lesion-based analyses to more thoroughly investigate the role of dual-tracer imaging in the heterogeneous landscape of NET disease.

## Conclusions

[^18^F]FDG PET and changes in metabolic status in patients undergoing PRRT play a key prognostic role. Assessing metabolic status before PRRT and during follow-up enables prediction of TTP and OS, even in patients considered for rechallenge PRRT. Standardizing the use of dual-tracer imaging in patients receiving PRRT is likely to be a valuable approach for improving clinical decision-making in patients with advanced metastatic NET.

## Supplementary Material

Supplementary figures and table.

## Figures and Tables

**Table 1:** Patients' characteristics

Characteristic	n
Total number of patients	100
Age at initial diagnosis (years)	
Median (Range)	54 (29 - 83)
Gender	
Male	64
Female	36
Primary tumor site	
Pancreas	43
Midgut	38
Unknown origin	6
Lung	5
Stomach	4
Colorectal	4
Sites of metastases	
Liver	93
Lymph nodes	40
Bone	30
Lung	7
Others	13
Grade	
1	16
2	66
3	18
Initial PRRT	
[^177^Lu]Lu-DOTA-TATE	78
[^90^Y]Y-DOTA-TOC	16
[^90^Y]Y-DOTA-TATE	3
[^90^Y]Y-DOTA-TATE+[^177^Lu]Lu-DOTA-TATE	2
[^90^Y]Y-DOTA-TOC+[^177^Lu]Lu-DOTA-TATE	1
Rechallenge PRRT	
[^177^Lu]Lu-DOTA-TATE	89
[^90^Y]Y-DOTA-TOC	9
[^90^Y]Y-DOTA-TATE	2
n=number of patients, except age in years	

**Table 2:** Univariate and multivariate Cox regression analysis for TTP.

Variable	Univariate Cox Regression			Multivariate Cox Regression		
	HR	95%CI	*p*	HR	95%CI	*p*
Age	0.998	0.982; 1.014	0.768			
Gender	0.967	0.640; 1.462	0.874			
Primary tumor site	1.157	0.775; 1.727	0.476	1.097	0.703; 1.711	0.684
Lymph node metastases	0.874	0.583; 1.311	0.515	0.986	0.638; 1.523	0.949
Bone metastases	1.861	1.189; 2.921	**0.007**	1.015	0.590; 1.746	0.956
Lung metastases	1.592	0.728; 3.480	0.244			
NET grade						
Grade 1 vs. 3	0.230	0.112; 0.471	**<0.001**	0.374	0.171; 0.820	**0.014**
Grade 2 vs. 3	0.338	0.195; 0.587	**<0.001**	0.448	0.250; 0.801	**0.007**
Grade 2 vs. 1	2.866	0.671; 12.239	0.155			
FDG status before 1^st^ PRRT	1.972	1.318; 2.951	**<0.001**	1.300	0.816; 2.073	0.270
FDG status after 1^st^ PRRT	18.921	9.068; 39.477	**<0.001**	15.549	7.060; 34.244	**<0.001**
FDG changes after 1^st^ PRRT						
No change vs. FDG^-^/FDG^+^	0.353	0.156; 0.799	**0.013**			
FDG^+^/FDG^-^ vs. FDG^-^/ FDG^+^	0.243	0.097; 0.607	**0.002**			
No change vs. FDG^+^/FDG^-^	0.687	0.409; 1.154	0.156			

Legend: CI = confidence interval; FDG = [^18^F]fluorodeoxyglucose; HR = hazard ratio; NET = neuroendocrine tumor; PET = positron emission tomography; PRRT = peptide receptor radionuclide therapy

**Table 3:** Univariate and multivariate Cox regression analysis for OS.

Variable	Univariate Cox Regression			Multivariate Cox Regression		
	HR	95%CI	*p*	HR	95%CI	*p*
Age	1.015	0.989; 1.043	0.263			
Gender	1.328	0.680; 2.593	0.407			
Primary	1.733	0.898; 3.342	0.101	1.258	0.532; 2.975	0.602
Lymph node metastases	0.533	0.261; 1.088	0.084	0.223	0.09; 0.552	**0.001**
Bone metastases	1.579	0.796; 3.131	0.191	0.561	0.233; 1.353	0.198
Lung metastases	2.036	0.715; 5.792	0.183			
NET grade						
Grade 1 vs. 3	0.123	0.035; 0.433	**0.001**	0.255	0.061; 1.074	0.062
Grade 2 vs. 3	0.207	0.101; 0.424	**<0.001**	0.521	0.229; 1.187	0.121
Grade 2 vs. 1	1.685	0.496; 5.723	0.403			
FDG status before 1^st^ PRRT	1.625	0.840; 3.143	0.149	1.014	0.386; 2.666	0.977
FDG status after 1^st^ PRRT	39.383	12.181; 127.334	**<0.001**	27.963	6.381; 122.533	**<0.001**
FDG changes after 1^st^ PRRT						
No change vs. FDG^-^/FDG^+^	0.167	0.064; 0.437	**<0.001**			
FDG^+^/FDG^-^ vs. FDG^-^/ FDG^+^	0.037	0.009; 0.163	**<0.001**			
FDG^+^/FDG^-^ vs. no change	0.224	0.067; 0.743	**0.015**			
FDG status after 2^nd^ PRRT	14.580	6.405; 33.192	**<0.001**	10.321	3.316; 32.128	**<0.001**
FDG changes after 2^nd^ PRRT						
No change vs. FDG^-^/FDG^+^	0.442	0.212; 0.921	**0.029**			
FDG^+^/FDG^-^ vs. FDG^-^/ FDG^+^	0.135	0.043; 0.424	**<0.001**			
FDG^+^/FDG^-^ vs. no change	0.306	0.104; 0.899	**0.031**			

Legend: CI = confidence interval; FDG = [^18^F]fluorodeoxyglucose; HR = hazard ratio; NET = neuroendocrine tumor; PET = positron emission tomography; PRRT = peptide receptor radionuclide therapy

## Abbreviations

CI: confidence interval
CR: complete remission
ENETS: European Neuroendocrine Tumor Society
ESMO: European Society for Medical Oncology
FDG: [18F]Fluorodeoxyglucose
GEP: gastroenteropancreatic
HR: hazard ratio
MRI: magnetic resonance imaging
MTV: metabolic tumor volume
NET: neuroendocrine tumors
OS: overall survival
PD: progressive disease
PET/CT: positron emission tomography/computed tomography
PR: partial remission
PRRT: peptide receptor radionuclide therapy
SD: stable disease
SSA: somatostatin analogs
SSTR: somatostatin receptors
TLG: total lesion glycolysis
TTP: time to progression
