# Supplementary material for: Prognostic implications of [¹⁸F]FDG PET and metabolic changes in patients with advanced metastatic neuroendocrine tumors undergoing rechallenge PRRT: final results from a multicenter 10-year survival WARMTH study
Source: Theranostics. 2026 Jan 1;16(3):1227–37. doi: 10.7150/thno.123273 (PMC12679270; doi:10.7150/thno.123273)
Supplement: Supplementary file 1 — Supplementary figures and table. [file thnov16p1227s1.pdf]

Prognostic implications of [^{18}F]FDG PET and metabolic changes in patients with advanced metastatic neuroendocrine tumors undergoing rechallenge PRRT: final results from a multicenter 10-year survival WARMTH study

Supplementary materials

Giulia Santo[1,2,#], Margarida Rodrigues[1,#], Diana Paez[3], Olga Morozova[3], Levent Kabasakal[4], Kalevi Kairemo[5], Chiara Maria Grana[6], Richard P. Baum[7,8], Gianpaolo di Santo[1], Irene J. Virgolini[1,*]

[1] Department of Nuclear Medicine, Medical University of Innsbruck, Innsbruck, Austria

[2] Department of Experimental and Clinical Medicine, "Magna Graecia" University of Catanzaro, Catanzaro, Italy

[3] Nuclear Medicine and Diagnostic Imaging Section, Division of Human Health, International Atomic Energy Agency (IAEA), Vienna, Austria

[4] Department of Nuclear Medicine, İstanbul University-Cerrahpasa Medical Faculty, İstanbul, Türkiye

[5] Nuclear Medicine and Theragnostics, Docrates Cancer Center, Helsinki, Finland

[6] Radiometabolic Therapy Unit, Division of Nuclear Medicine, IRCCS IEO European Institute of Oncology, Milano, Italy

[7] Molecular Radiotherapy, Curanosticum Wiesbaden-Frankfurt, Germany

[8] Theranostics Center for Molecular Radiotherapy and Precision Oncology, ENETS Center of Excellence, Zentralklinik Bad Berka, Bad Berka, Germany

Corresponding author:

Irene J. Virgolini

Department of Nuclear Medicine, Medical University of Innsbruck,

Anichstrasse 35, 6020 Innsbruck, Austria

Tel: +43-512-504-22651, fax: +43-512-504-22659

e-mail: irene.virgolini@tirol-kliniken.at

First authors:

Giulia Santo and Margarida Rodrigues share the first authorship

Department of Nuclear Medicine, Medical University of Innsbruck,

Anichstrasse 35, 6020 Innsbruck, Austria

Supplementary Table

Table S1. Information on PET/CT scanners, the administered activity of radiopharmaceuticals, the main acquisition parameters and the software used for imaging review at each center.

**Institution**	**Manufacturer**	**Model**	**Administered activity**	**Acquisition parameters**	**Software for imaging revision**
Medical University of Innsbruck, Innsbruck, Austria	GE HealthCare	MS-Advance or MS-Discovery 450	100–150 MBq of [^{68}Ga]Ga-DOTATOC;<br>200–300 MBq of [^{18}F]FDG	Images were acquired from the skull to the mid-thigh. Attenuation correction was performed using transmission data obtained with a ^{67}Ge pin source at 3 min per bed position (MS-Advance) or a CT scan (MS-Discovery 450). Ordered-subsets expectation maximization was used for image reconstruction.	eNTEGRA, GE HealthCare
İstanbul University-Cerrahpasa Medical Faculty, İstanbul, Türkiye	Siemens, Knoxville, TN, USA	Biograph LSO HI-REZ PET/CT	110–200 MBq (mean 143.19±34.04 MBq) of [^{68}Ga]Ga-DOTATATE;<br>370–703 MBq of [^{18}F]FDG	A CT topogram was first acquired to define the axial range of the PET/CT study, covering the area from the skull to the mid-thighs. After that, a CT transmission scan without i.v. contrast enhancement was acquired with low tube current (130 kVp, 48–76 mAs), a slice thickness of 4.0 mm, 0.6 s gantry rotation, and a collimator width of 6×3 mm. Then PET emission scanning with a duration of 3 min per bed position was performed. For attenuation correction, CT transmission images were used, and an iterative method was used for image reconstruction.	advantageWorkstation version: 4.6; GE Healthcare
Docrates Cancer Center, Helsinki, Finland	Siemens Healthineers, Erlangen, Germany	TruePoint PET-CT Siemens Biograph 6	120–170 MBq of [^{68}Ga]Ga-DOTATOC;<br>210–310 MBq of [^{18}F]FDG	Whole body imaging covered the area from the calvarium to the mid-thighs. Standard vendor-provided reconstruction algorithms were used to reconstruct PET images. CT images were taken using 3.75 mm slice thickness. Attenuation- and nonattenuation-corrected datasets were reconstructed, and the images were analyzed using syngo.via software (later with MIM software).	syngo.via, Siemens Healthineers
IRCCS IEO European Institute of Oncology, Milano, Italy	GE HealthCare	Discovery STE scanner	3.0 MBq/kg of [^{68}Ga]Ga-DOTATOC;<br>2.5 MBq/Kg of [^{18}F]FDG	Approximately 60 minutes after injection, static PET/CT imaging in 3D covering the upper torso from eyebrows to midthighs (3-minute emission scan/position) started. Attenuation correction was performed using a low-dose ultra-fast CT protocol (80 mAs, 140 kV, 0.3 mSv/FOV). Images were reconstructed using 3D OSEM iterative reconstruction algorithm (2 iterations, 28 subsets) with a 6.0 mm FWHM postfilter.	Xeleris GEHC
Theranostics Center for Molecular Radiotherapy and Precision Oncology, Zentralklinik Bad Berka, Bad Berka, Germany.	Siemens Medical Solutions AG, Erlangen, Germany	Biograph Duo or Biograph mCT Flow 64	46–260 MBq of [^{68}Ga]Ga-DOTANOC, [^{68}Ga]Ga-DOTATOC, or [^{68}Ga]Ga-DOTATATE;<br>350–600 MBq of [^{18}F]FDG	Approximately 45–90 min after the intravenous injection of ^{68}Ga-DOTA-NOC, ^{68}Ga-DOTATOC, or ^{68}Ga-DOTATATE and 45–90 min after the intravenous injection of 350–600 MBq of ^{18}F-FDG, respectively, PET/CT images were acquired from the skull to the middle part of the thigh. Contrast-enhanced CT (spiral CT using a Biograph mCT Flow 64) was acquired after the intravenous administration of 60–100 mL of nonionic iodinated contrast agent.	syngo.via, Siemens Healthineers

Supplementary Figures

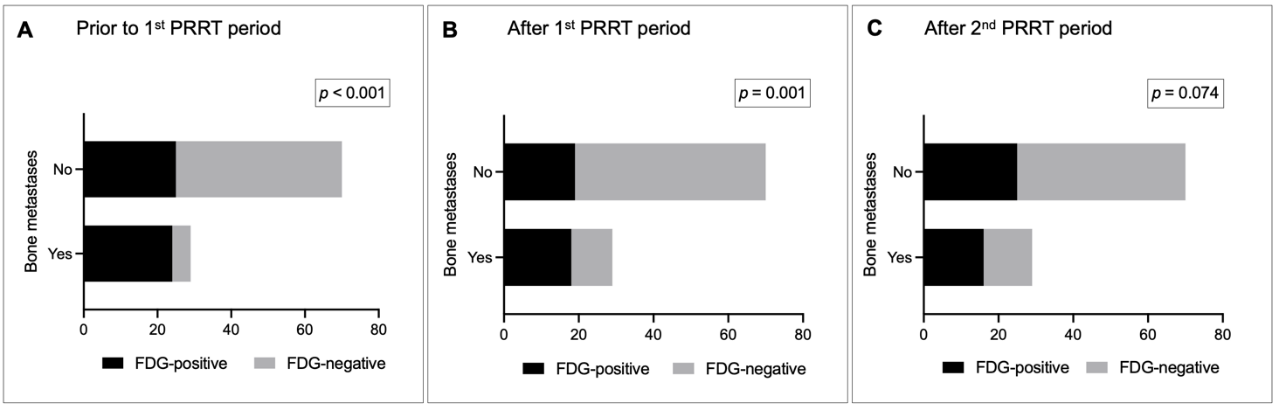

Figure S1. Relationship between the presence of bone metastases and FDG status (A) prior to the 1st PRRT period, (B) after the 1st PRRT period, and (C) after the 2nd PRRT period

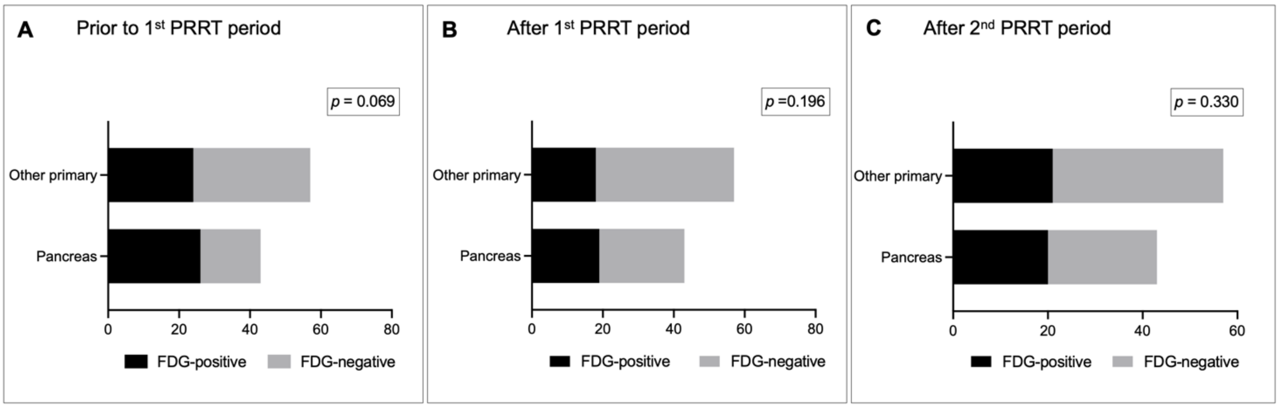

Figure S2. Relationship between primary tumor site (other primary vs. pancreatic NET) and FDG status (A) prior to the 1st PRRT period, (B) after the 1st PRRT period, and (C) after the 2nd PRRT period

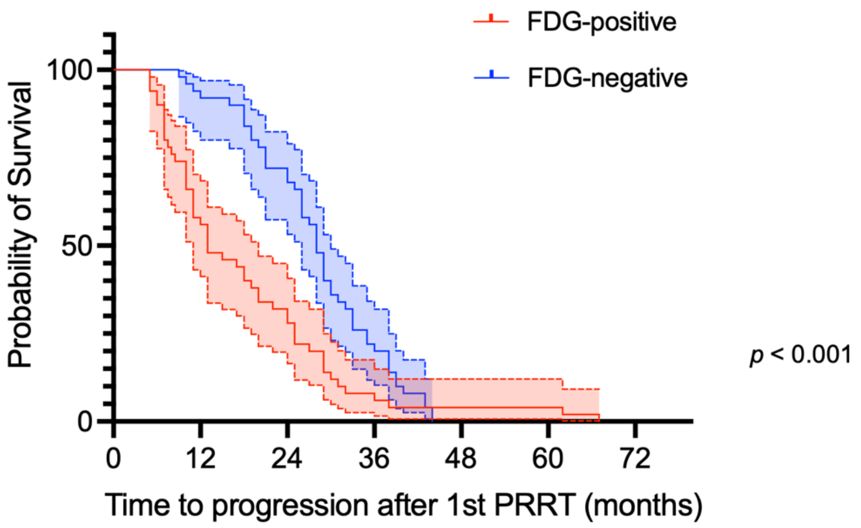

Median TTP
FDG positive: 13 months (95% CI: 7.2 – 18.8 months)
FDG negative: 28 months (95% CI: 26.3 – 29.7 months)

Figure S3. Kaplan-Meier curve for time to progression (TTP) stratified by a) [^{18}F]fluorodeoxyglucose (FDG) status before the first peptide receptor radionuclide therapy (PRRT) period
